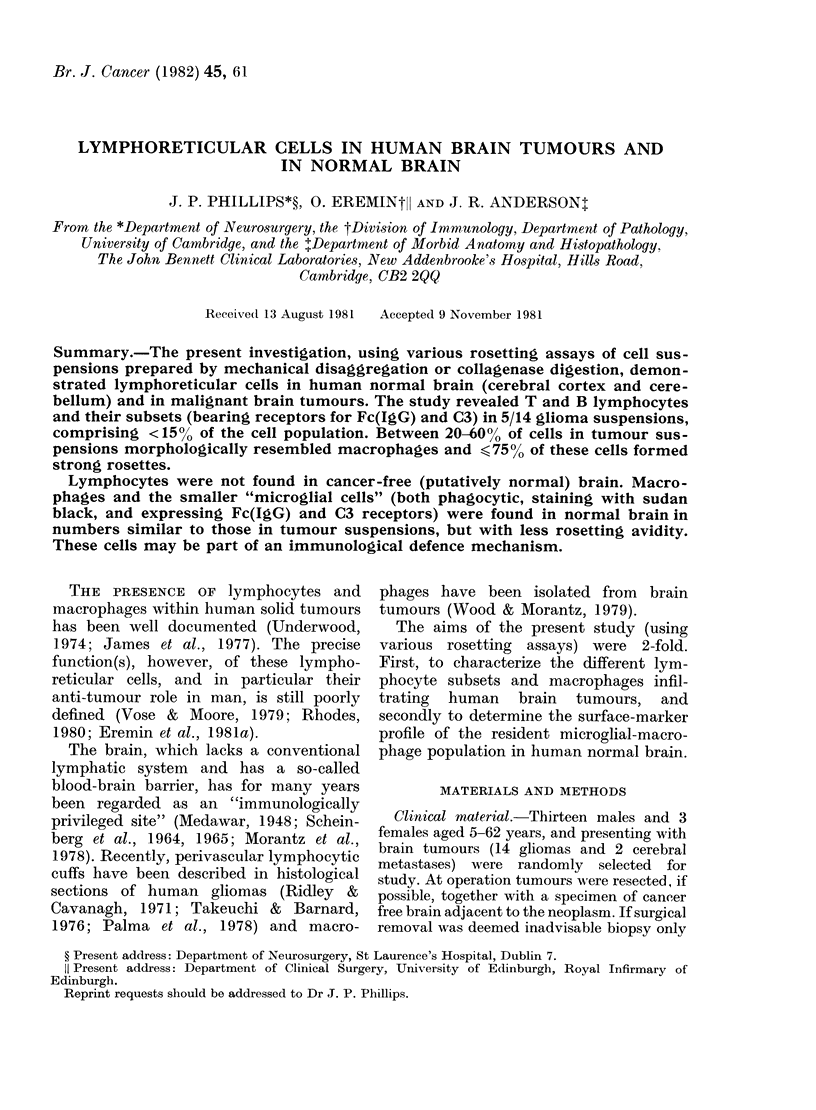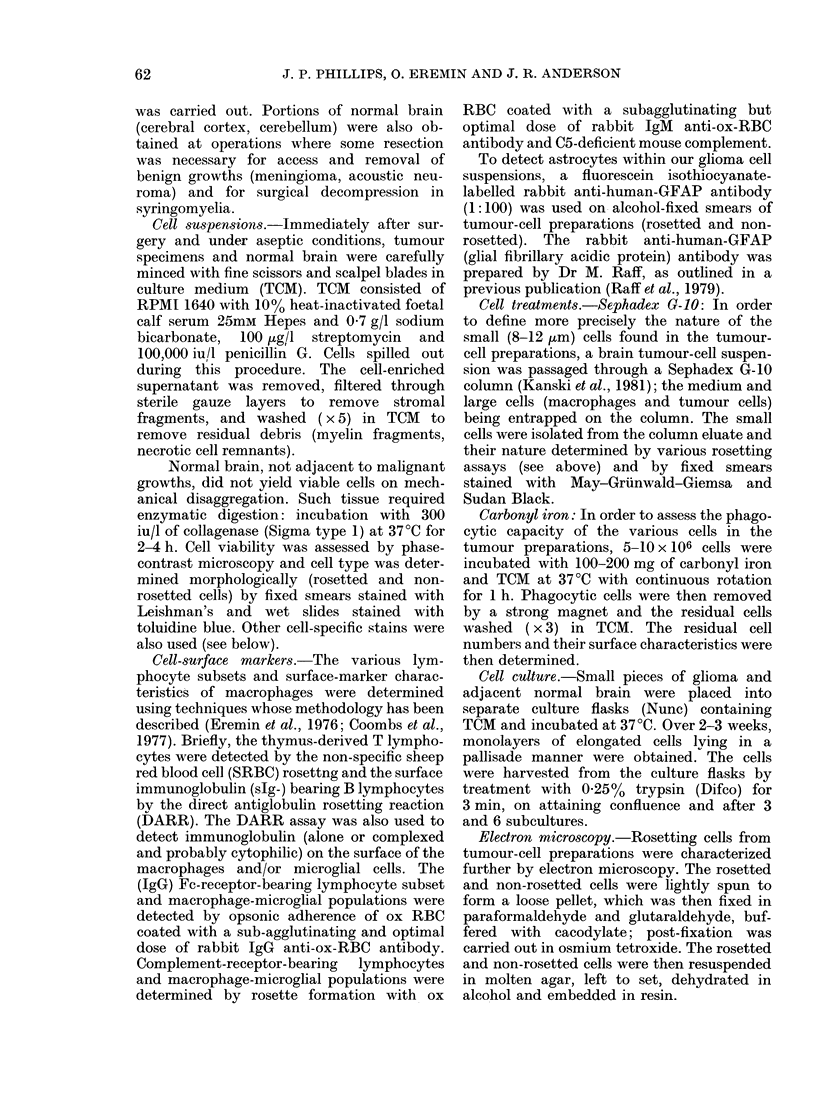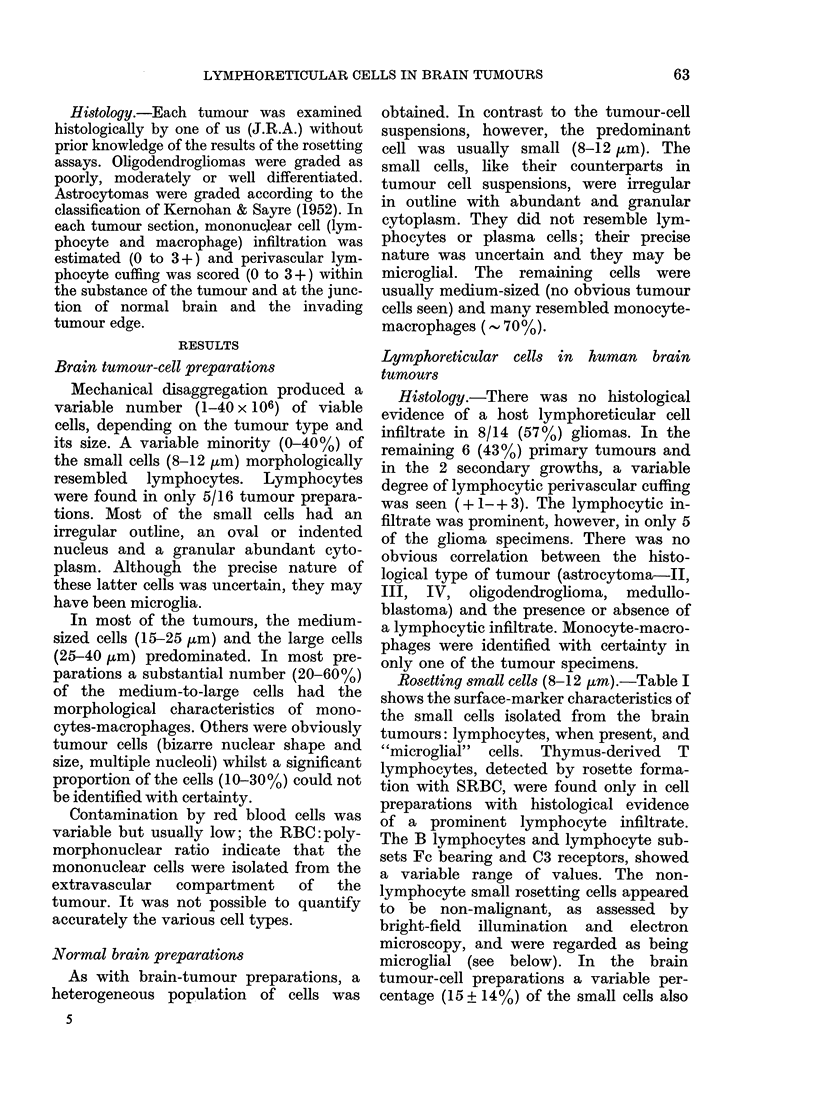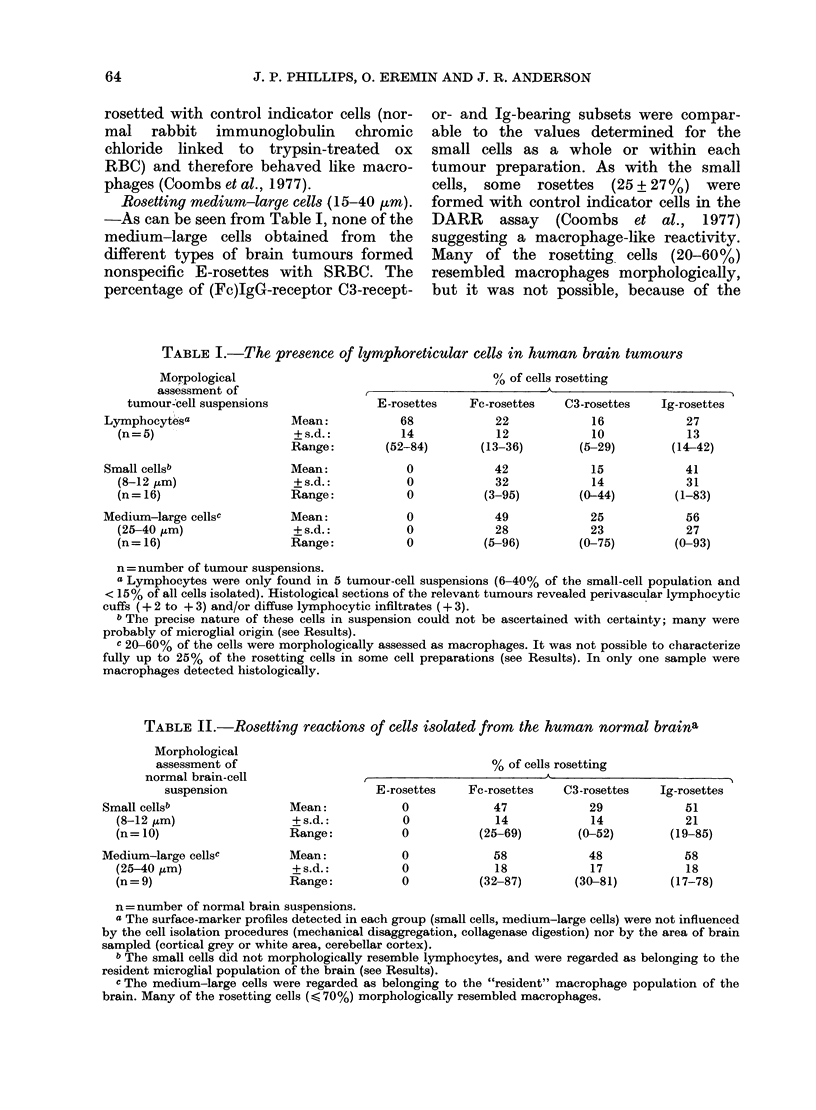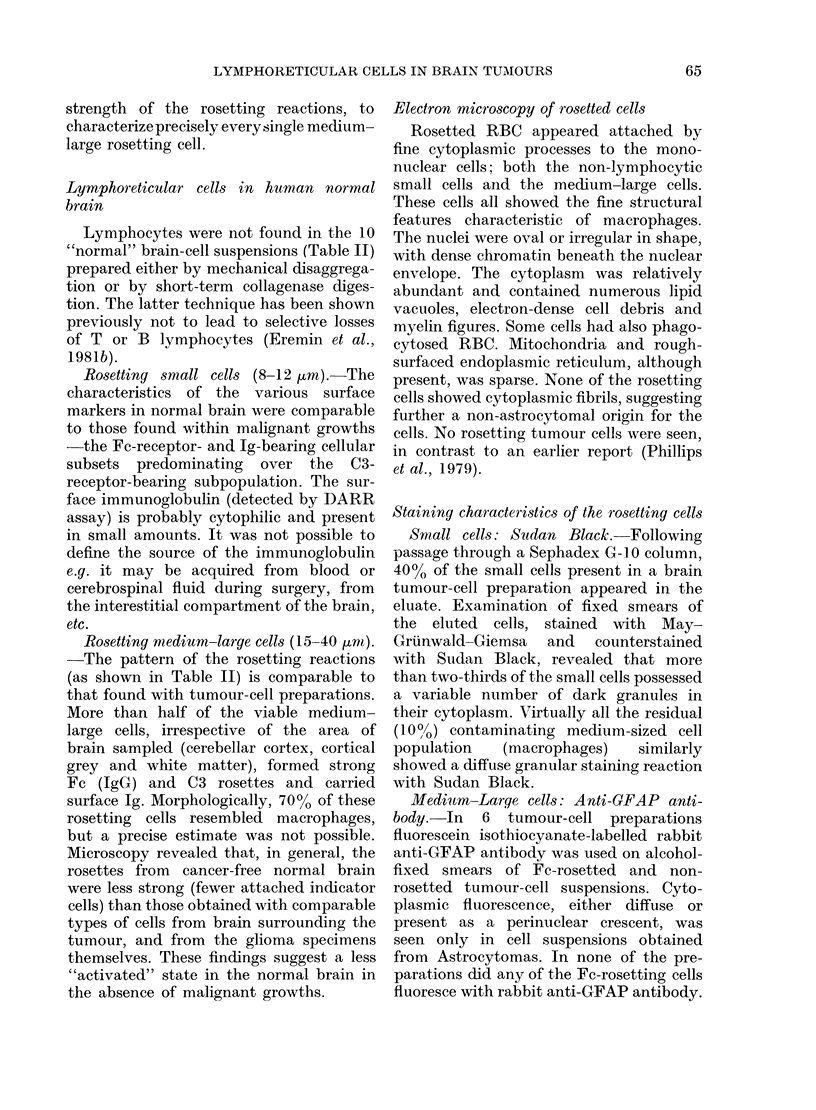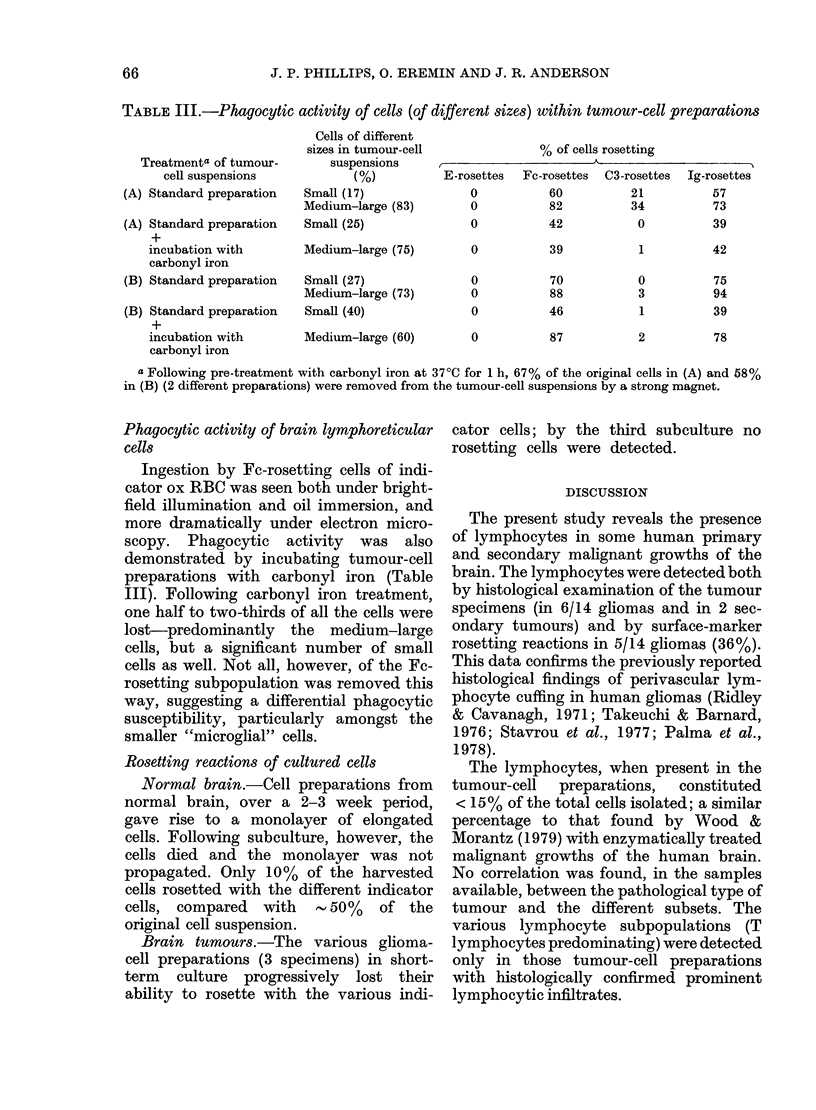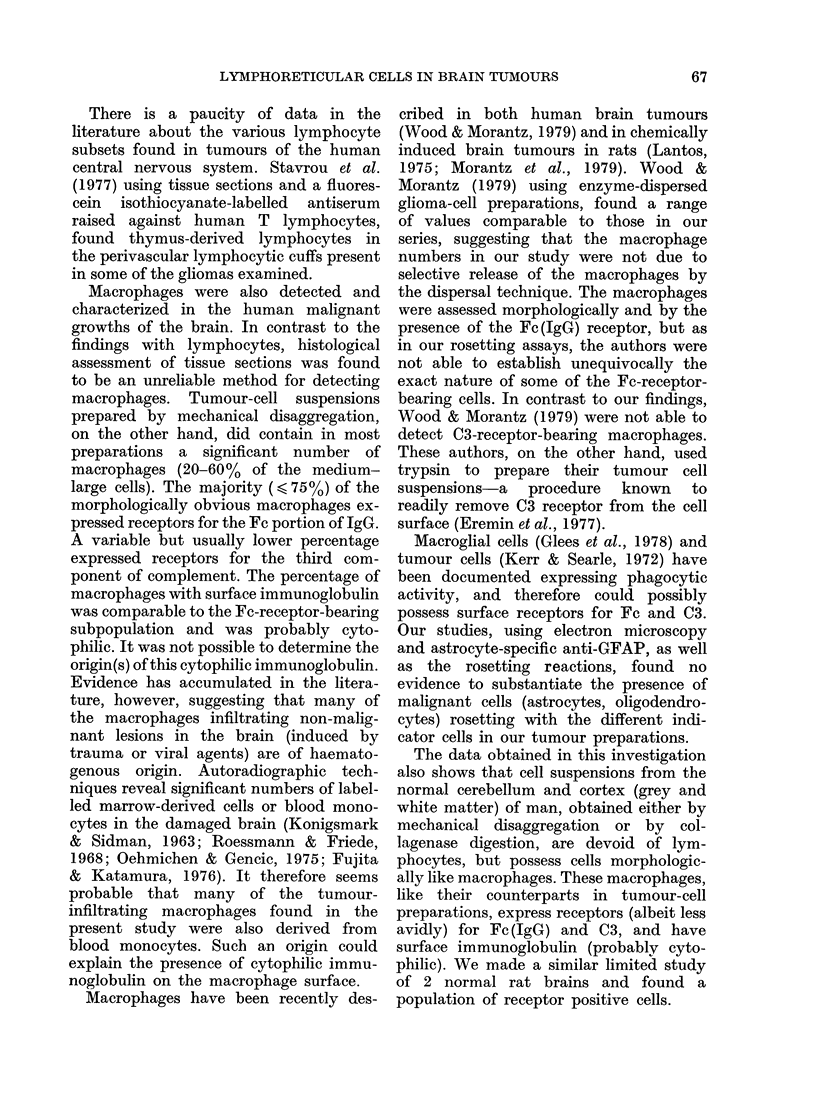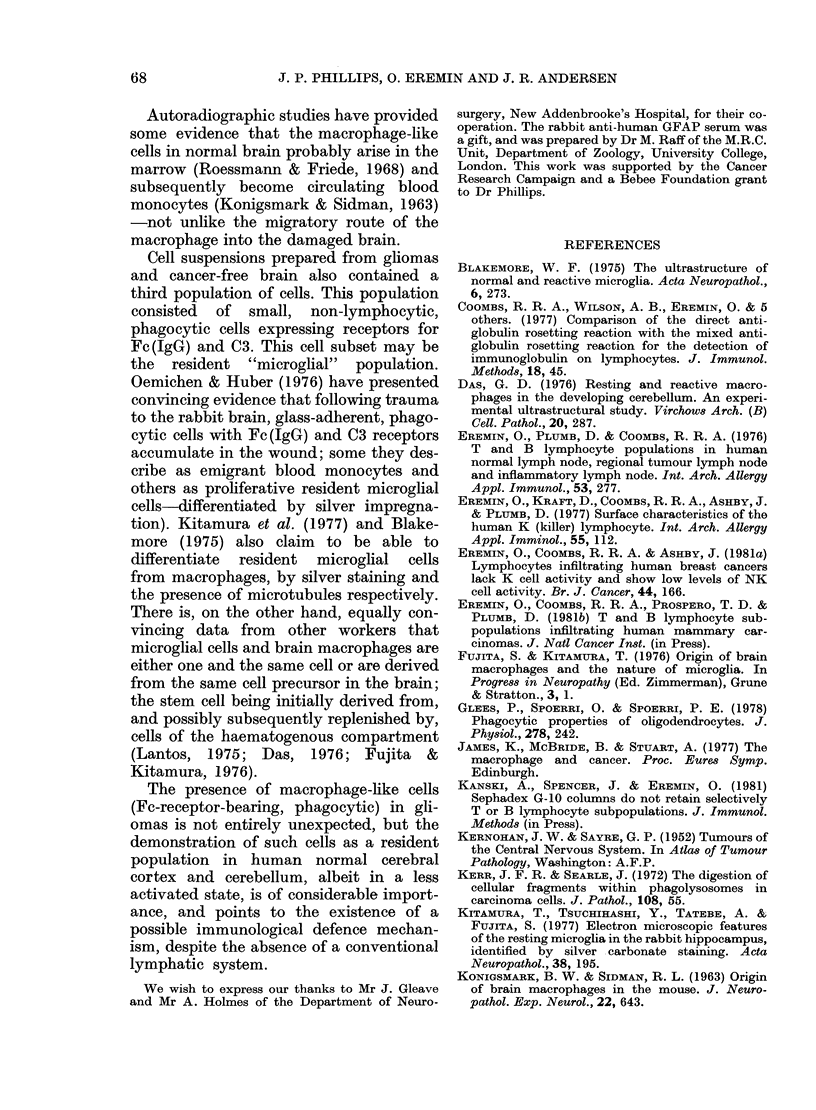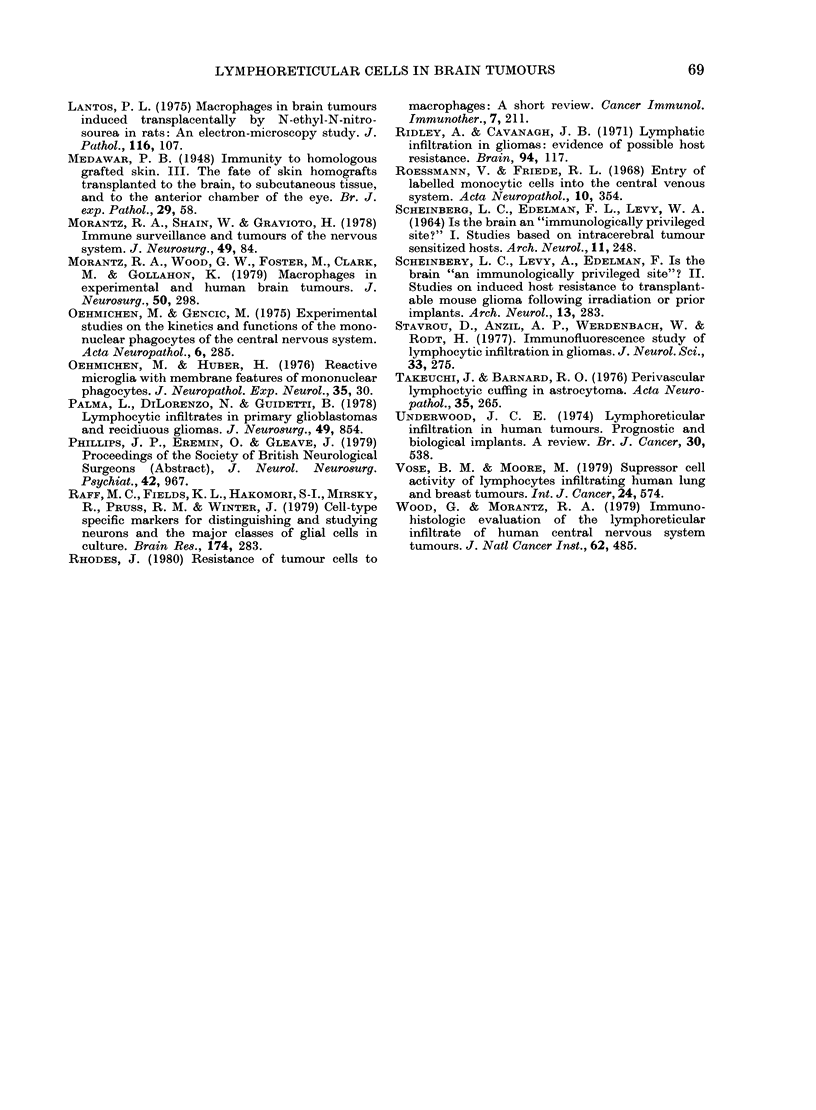# Lymphoreticular cells in human brain tumours and in normal brain.

**DOI:** 10.1038/bjc.1982.8

**Published:** 1982-01

**Authors:** J. P. Phillips, O. Eremin, J. R. Anderson

## Abstract

The present investigation, using various rosetting assays of cell suspensions prepared by mechanical disaggregation or collagenase digestion, demonstrated lymphoreticular cells in human normal brain (cerebral cortex and cerebellum) and in malignant brain tumours. The study revealed T and B lymphocytes and their subsets (bearing receptors for Fc(IgG) and C3) in 5/14 glioma suspensions, comprising less than 15% of the cell population. Between 20-60% of cells in tumour suspensions morphologically resembled macrophages and less than or equal to 75% of these cells formed strong rosettes. Lymphocytes were not found in cancer-free (putatively normal) brain. Macrophages and the smaller "microglial cells" (both phagocytic, staining with sudan black, and expressing Fc(IgG) and C3 receptors) were found in normal brain in numbers similar to those in tumour suspensions, but with less rosetting avidity. These cells may be part of an immunological defence mechanism.


*Br. J. Cancer* (1982) **45**, 61

### LYMPHORETICULAR CELLS IN HUMAN BRAIN TUMOURS AND IN NORMAL BRAIN

J. P. PHILLIPS*§, O. EREMIN†‖ AND J. R. ANDERSON‡

*From the *Department of Neurosurgery, the †Division of Immunology, Department of Pathology, University of Cambridge, and the ‡Department of Morbid Anatomy and Histopathology, The John Bennett Clinical Laboratories, New Addenbrooke's Hospital, Hills Road, Cambridge, CB2 2QQ*

Received 13 August 1981 Accepted 9 November 1981

**Summary.—The present investigation, using various rosetting assays of cell suspensions prepared by mechanical disaggregation or collagenase digestion, demonstrated lymphoreticular cells in human normal brain (cerebral cortex and cerebellum) and in malignant brain tumours. The study revealed T and B lymphocytes and their subsets (bearing receptors for Fc(IgG) and C3) in 5/14 glioma suspensions, comprising <15% of the cell population. Between 20–60% of cells in tumour suspensions morphologically resembled macrophages and ≤75% of these cells formed strong rosettes.**

**Lymphocytes were not found in cancer-free (putatively normal) brain. Macrophages and the smaller "microglial cells" (both phagocytic, staining with sudan black, and expressing Fc(IgG) and C3 receptors) were found in normal brain in numbers similar to those in tumour suspensions, but with less rosetting avidity. These cells may be part of an immunological defence mechanism.**

THE PRESENCE OF lymphocytes and macrophages within human solid tumours has been well documented (Underwood, 1974; James *et al.*, 1977). The precise function(s), however, of these lymphoreticular cells, and in particular their anti-tumour role in man, is still poorly defined (Vose & Moore, 1979; Rhodes, 1980; Eremin *et al.*, 1981*a*).

The brain, which lacks a conventional lymphatic system and has a so-called blood-brain barrier, has for many years been regarded as an "immunologically privileged site" (Medawar, 1948; Scheinberg *et al.*, 1964, 1965; Morantz *et al.*, 1978). Recently, perivascular lymphocytic cuffs have been described in histological sections of human gliomas (Ridley & Cavanagh, 1971; Takeuchi & Barnard, 1976; Palma *et al.*, 1978) and macrophages have been isolated from brain tumours (Wood & Morantz, 1979).

The aims of the present study (using various rosetting assays) were 2-fold. First, to characterize the different lymphocyte subsets and macrophages infiltrating human brain tumours, and secondly to determine the surface-marker profile of the resident microglial-macrophage population in human normal brain.

### MATERIALS AND METHODS

*Clinical material.*—Thirteen males and 3 females aged 5–62 years, and presenting with brain tumours (14 gliomas and 2 cerebral metastases) were randomly selected for study. At operation tumours were resected, if possible, together with a specimen of cancer free brain adjacent to the neoplasm. If surgical removal was deemed inadvisable biopsy only

§ Present address: Department of Neurosurgery, St Laurence's Hospital, Dublin 7.

‖ Present address: Department of Clinical Surgery, University of Edinburgh, Royal Infirmary of Edinburgh.

Reprint requests should be addressed to Dr J. P. Phillips.

was carried out. Portions of normal brain (cerebral cortex, cerebellum) were also obtained at operations where some resection was necessary for access and removal of benign growths (meningioma, acoustic neuroma) and for surgical decompression in syringomyelia.

*Cell suspensions.*—Immediately after surgery and under aseptic conditions, tumour specimens and normal brain were carefully minced with fine scissors and scalpel blades in culture medium (TCM). TCM consisted of RPMI 1640 with 10% heat-inactivated foetal calf serum 25mM Hepes and 0·7 g/l sodium bicarbonate, 100 μg/l streptomycin and 100,000 iu/l penicillin G. Cells spilled out during this procedure. The cell-enriched supernatant was removed, filtered through sterile gauze layers to remove stromal fragments, and washed (×5) in TCM to remove residual debris (myelin fragments, necrotic cell remnants).

Normal brain, not adjacent to malignant growths, did not yield viable cells on mechanical disaggregation. Such tissue required enzymatic digestion: incubation with 300 iu/l of collagenase (Sigma type 1) at 37°C for 2–4 h. Cell viability was assessed by phase-contrast microscopy and cell type was determined morphologically (rosetted and non-rosetted cells) by fixed smears stained with Leishman's and wet slides stained with toluidine blue. Other cell-specific stains were also used (see below).

*Cell-surface markers.*—The various lymphocyte subsets and surface-marker characteristics of macrophages were determined using techniques whose methodology has been described (Eremin *et al.*, 1976; Coombs *et al.*, 1977). Briefly, the thymus-derived T lymphocytes were detected by the non-specific sheep red blood cell (SRBC) rosettng and the surface immunoglobulin (sIg-) bearing B lymphocytes by the direct antiglobulin rosetting reaction (DARR). The DARR assay was also used to detect immunoglobulin (alone or complexed and probably cytophilic) on the surface of the macrophages and/or microglial cells. The (IgG) Fc-receptor-bearing lymphocyte subset and macrophage-microglial populations were detected by opsonic adherence of ox RBC coated with a sub-agglutinating and optimal dose of rabbit IgG anti-ox-RBC antibody. Complement-receptor-bearing lymphocytes and macrophage-microglial populations were determined by rosette formation with ox RBC coated with a subagglutinating but optimal dose of rabbit IgM anti-ox-RBC antibody and C5-deficient mouse complement.

To detect astrocytes within our glioma cell suspensions, a fluorescein isothiocyanate-labelled rabbit anti-human-GFAP antibody (1:100) was used on alcohol-fixed smears of tumour-cell preparations (rosetted and non-rosetted). The rabbit anti-human-GFAP (glial fibrillary acidic protein) antibody was prepared by Dr M. Raff, as outlined in a previous publication (Raff *et al.*, 1979).

*Cell treatments.*—*Sephadex G-10*: In order to define more precisely the nature of the small (8–12 μm) cells found in the tumour-cell preparations, a brain tumour-cell suspension was passaged through a Sephadex G-10 column (Kanski *et al.*, 1981); the medium and large cells (macrophages and tumour cells) being entrapped on the column. The small cells were isolated from the column eluate and their nature determined by various rosetting assays (see above) and by fixed smears stained with May–Grünwald–Giemsa and Sudan Black.

*Carbonyl iron:* In order to assess the phagocytic capacity of the various cells in the tumour preparations, 5–10 × $10^6$ cells were incubated with 100–200 mg of carbonyl iron and TCM at 37°C with continuous rotation for 1 h. Phagocytic cells were then removed by a strong magnet and the residual cells washed (×3) in TCM. The residual cell numbers and their surface characteristics were then determined.

*Cell culture.*—Small pieces of glioma and adjacent normal brain were placed into separate culture flasks (Nunc) containing TCM and incubated at 37°C. Over 2–3 weeks, monolayers of elongated cells lying in a pallisade manner were obtained. The cells were harvested from the culture flasks by treatment with 0·25% trypsin (Difco) for 3 min, on attaining confluence and after 3 and 6 subcultures.

*Electron microscopy.*—Rosetting cells from tumour-cell preparations were characterized further by electron microscopy. The rosetted and non-rosetted cells were lightly spun to form a loose pellet, which was then fixed in paraformaldehyde and glutaraldehyde, buffered with cacodylate; post-fixation was carried out in osmium tetroxide. The rosetted and non-rosetted cells were then resuspended in molten agar, left to set, dehydrated in alcohol and embedded in resin.

*Histology*.—Each tumour was examined histologically by one of us (J.R.A.) without prior knowledge of the results of the rosetting assays. Oligodendrogliomas were graded as poorly, moderately or well differentiated. Astrocytomas were graded according to the classification of Kernohan & Sayre (1952). In each tumour section, mononuclear cell (lymphocyte and macrophage) infiltration was estimated (0 to 3+) and perivascular lymphocyte cuffing was scored (0 to 3+) within the substance of the tumour and at the junction of normal brain and the invading tumour edge.

RESULTS

*Brain tumour-cell preparations*

Mechanical disaggregation produced a variable number ($1–40 \times 10^6$) of viable cells, depending on the tumour type and its size. A variable minority (0–40%) of the small cells (8–12 $\mu$m) morphologically resembled lymphocytes. Lymphocytes were found in only 5/16 tumour preparations. Most of the small cells had an irregular outline, an oval or indented nucleus and a granular abundant cytoplasm. Although the precise nature of these latter cells was uncertain, they may have been microglia.

In most of the tumours, the medium-sized cells (15–25 $\mu$m) and the large cells (25–40 $\mu$m) predominated. In most preparations a substantial number (20–60%) of the medium-to-large cells had the morphological characteristics of monocytes-macrophages. Others were obviously tumour cells (bizarre nuclear shape and size, multiple nucleoli) whilst a significant proportion of the cells (10–30%) could not be identified with certainty.

Contamination by red blood cells was variable but usually low; the RBC:polymorphonuclear ratio indicate that the mononuclear cells were isolated from the extravascular compartment of the tumour. It was not possible to quantify accurately the various cell types.

*Normal brain preparations*

As with brain-tumour preparations, a heterogeneous population of cells was obtained. In contrast to the tumour-cell suspensions, however, the predominant cell was usually small (8–12 $\mu$m). The small cells, like their counterparts in tumour cell suspensions, were irregular in outline with abundant and granular cytoplasm. They did not resemble lymphocytes or plasma cells; their precise nature was uncertain and they may be microglial. The remaining cells were usually medium-sized (no obvious tumour cells seen) and many resembled monocyte-macrophages ($\sim$70%).

*Lymphoreticular cells in human brain tumours*

*Histology*.—There was no histological evidence of a host lymphoreticular cell infiltrate in 8/14 (57%) gliomas. In the remaining 6 (43%) primary tumours and in the 2 secondary growths, a variable degree of lymphocytic perivascular cuffing was seen (+1–+3). The lymphocytic infiltrate was prominent, however, in only 5 of the glioma specimens. There was no obvious correlation between the histological type of tumour (astrocytoma—II, III, IV, oligodendroglioma, medulloblastoma) and the presence or absence of a lymphocytic infiltrate. Monocyte-macrophages were identified with certainty in only one of the tumour specimens.

*Rosetting small cells* (8–12 $\mu$*m*).—Table I shows the surface-marker characteristics of the small cells isolated from the brain tumours: lymphocytes, when present, and "microglial" cells. Thymus-derived T lymphocytes, detected by rosette formation with SRBC, were found only in cell preparations with histological evidence of a prominent lymphocyte infiltrate. The B lymphocytes and lymphocyte subsets Fc bearing and C3 receptors, showed a variable range of values. The non-lymphocyte small rosetting cells appeared to be non-malignant, as assessed by bright-field illumination and electron microscopy, and were regarded as being microglial (see below). In the brain tumour-cell preparations a variable percentage (15 ± 14%) of the small cells also rosetted with control indicator cells (normal rabbit immunoglobulin chromic chloride linked to trypsin-treated ox RBC) and therefore behaved like macrophages (Coombs *et al.*, 1977).

*Rosetting medium–large cells* (15–40 *μm*). —As can be seen from Table I, none of the medium–large cells obtained from the different types of brain tumours formed nonspecific E-rosettes with SRBC. The percentage of (Fc)IgG-receptor C3-receptor- and Ig-bearing subsets were comparable to the values determined for the small cells as a whole or within each tumour preparation. As with the small cells, some rosettes (25 ± 27%) were formed with control indicator cells in the DARR assay (Coombs *et al.*, 1977) suggesting a macrophage-like reactivity. Many of the rosetting cells (20–60%) resembled macrophages morphologically, but it was not possible, because of the

TABLE I.—*The presence of lymphoreticular cells in human brain tumours*

| Morphological assessment of tumour-cell suspensions | | % of cells rosetting: E-rosettes | Fc-rosettes | C3-rosettes | Ig-rosettes |
|---|---|---|---|---|---|
| Lymphocytes[a] | Mean: | 68 | 22 | 16 | 27 |
| (n = 5) | ± s.d.: | 14 | 12 | 10 | 13 |
| | Range: | (52–84) | (13–36) | (5–29) | (14–42) |
| Small cells[b] | Mean: | 0 | 42 | 15 | 41 |
| (8–12 μm) | ± s.d.: | 0 | 32 | 14 | 31 |
| (n = 16) | Range: | 0 | (3–95) | (0–44) | (1–83) |
| Medium–large cells[c] | Mean: | 0 | 49 | 25 | 56 |
| (25–40 μm) | ± s.d.: | 0 | 28 | 23 | 27 |
| (n = 16) | Range: | 0 | (5–96) | (0–75) | (0–93) |

n = number of tumour suspensions.

[a] Lymphocytes were only found in 5 tumour-cell suspensions (6–40% of the small-cell population and < 15% of all cells isolated). Histological sections of the relevant tumours revealed perivascular lymphocytic cuffs (+2 to +3) and/or diffuse lymphocytic infiltrates (+3).

[b] The precise nature of these cells in suspension could not be ascertained with certainty; many were probably of microglial origin (see Results).

[c] 20–60% of the cells were morphologically assessed as macrophages. It was not possible to characterize fully up to 25% of the rosetting cells in some cell preparations (see Results). In only one sample were macrophages detected histologically.

TABLE II.—*Rosetting reactions of cells isolated from the human normal brain*[a]

| Morphological assessment of normal brain-cell suspension | | % of cells rosetting: E-rosettes | Fc-rosettes | C3-rosettes | Ig-rosettes |
|---|---|---|---|---|---|
| Small cells[b] | Mean: | 0 | 47 | 29 | 51 |
| (8–12 μm) | ± s.d.: | 0 | 14 | 14 | 21 |
| (n = 10) | Range: | 0 | (25–69) | (0–52) | (19–85) |
| Medium–large cells[c] | Mean: | 0 | 58 | 48 | 58 |
| (25–40 μm) | ± s.d.: | 0 | 18 | 17 | 18 |
| (n = 9) | Range: | 0 | (32–87) | (30–81) | (17–78) |

n = number of normal brain suspensions.

[a] The surface-marker profiles detected in each group (small cells, medium–large cells) were not influenced by the cell isolation procedures (mechanical disaggregation, collagenase digestion) nor by the area of brain sampled (cortical grey or white area, cerebellar cortex).

[b] The small cells did not morphologically resemble lymphocytes, and were regarded as belonging to the resident microglial population of the brain (see Results).

[c] The medium–large cells were regarded as belonging to the "resident" macrophage population of the brain. Many of the rosetting cells (≤ 70%) morphologically resembled macrophages.

strength of the rosetting reactions, to characterize precisely every single medium–large rosetting cell.

*Lymphoreticular cells in human normal brain*

Lymphocytes were not found in the 10 "normal" brain-cell suspensions (Table II) prepared either by mechanical disaggregation or by short-term collagenase digestion. The latter technique has been shown previously not to lead to selective losses of T or B lymphocytes (Eremin *et al.*, 1981*b*).

*Rosetting small cells* (8–12 $\mu m$).—The characteristics of the various surface markers in normal brain were comparable to those found within malignant growths —the Fc-receptor- and Ig-bearing cellular subsets predominating over the C3-receptor-bearing subpopulation. The surface immunoglobulin (detected by DARR assay) is probably cytophilic and present in small amounts. It was not possible to define the source of the immunoglobulin *e.g.* it may be acquired from blood or cerebrospinal fluid during surgery, from the interestitial compartment of the brain, *etc.*

*Rosetting medium–large cells* (15–40 $\mu m$). —The pattern of the rosetting reactions (as shown in Table II) is comparable to that found with tumour-cell preparations. More than half of the viable medium–large cells, irrespective of the area of brain sampled (cerebellar cortex, cortical grey and white matter), formed strong Fc (IgG) and C3 rosettes and carried surface Ig. Morphologically, 70% of these rosetting cells resembled macrophages, but a precise estimate was not possible. Microscopy revealed that, in general, the rosettes from cancer-free normal brain were less strong (fewer attached indicator cells) than those obtained with comparable types of cells from brain surrounding the tumour, and from the glioma specimens themselves. These findings suggest a less "activated" state in the normal brain in the absence of malignant growths.

*Electron microscopy of rosetted cells*

Rosetted RBC appeared attached by fine cytoplasmic processes to the mononuclear cells; both the non-lymphocytic small cells and the medium–large cells. These cells all showed the fine structural features characteristic of macrophages. The nuclei were oval or irregular in shape, with dense chromatin beneath the nuclear envelope. The cytoplasm was relatively abundant and contained numerous lipid vacuoles, electron-dense cell debris and myelin figures. Some cells had also phagocytosed RBC. Mitochondria and rough-surfaced endoplasmic reticulum, although present, was sparse. None of the rosetting cells showed cytoplasmic fibrils, suggesting further a non-astrocytomal origin for the cells. No rosetting tumour cells were seen, in contrast to an earlier report (Phillips *et al.*, 1979).

*Staining characteristics of the rosetting cells*

*Small cells: Sudan Black.*—Following passage through a Sephadex G-10 column, 40% of the small cells present in a brain tumour-cell preparation appeared in the eluate. Examination of fixed smears of the eluted cells, stained with May–Grünwald–Giemsa and counterstained with Sudan Black, revealed that more than two-thirds of the small cells possessed a variable number of dark granules in their cytoplasm. Virtually all the residual (10%) contaminating medium-sized cell population (macrophages) similarly showed a diffuse granular staining reaction with Sudan Black.

*Medium–Large cells: Anti-GFAP antibody.*—In 6 tumour-cell preparations fluorescein isothiocyanate-labelled rabbit anti-GFAP antibody was used on alcohol-fixed smears of Fc-rosetted and non-rosetted tumour-cell suspensions. Cytoplasmic fluorescence, either diffuse or present as a perinuclear crescent, was seen only in cell suspensions obtained from Astrocytomas. In none of the preparations did any of the Fc-rosetting cells fluoresce with rabbit anti-GFAP antibody.

TABLE III.—*Phagocytic activity of cells (of different sizes) within tumour-cell preparations*

| Treatment[a] of tumour-cell suspensions | Cells of different sizes in tumour-cell suspensions (%) | % of cells rosetting | | | |
|---|---|---|---|---|---|
| | | E-rosettes | Fc-rosettes | C3-rosettes | Ig-rosettes |
| (A) Standard preparation | Small (17) | 0 | 60 | 21 | 57 |
| | Medium–large (83) | 0 | 82 | 34 | 73 |
| (A) Standard preparation + | Small (25) | 0 | 42 | 0 | 39 |
| incubation with carbonyl iron | Medium–large (75) | 0 | 39 | 1 | 42 |
| (B) Standard preparation | Small (27) | 0 | 70 | 0 | 75 |
| | Medium–large (73) | 0 | 88 | 3 | 94 |
| (B) Standard preparation + | Small (40) | 0 | 46 | 1 | 39 |
| incubation with carbonyl iron | Medium–large (60) | 0 | 87 | 2 | 78 |

[a] Following pre-treatment with carbonyl iron at 37°C for 1 h, 67% of the original cells in (A) and 58% in (B) (2 different preparations) were removed from the tumour-cell suspensions by a strong magnet.

*Phagocytic activity of brain lymphoreticular cells*

Ingestion by Fc-rosetting cells of indicator ox RBC was seen both under bright-field illumination and oil immersion, and more dramatically under electron microscopy. Phagocytic activity was also demonstrated by incubating tumour-cell preparations with carbonyl iron (Table III). Following carbonyl iron treatment, one half to two-thirds of all the cells were lost—predominantly the medium–large cells, but a significant number of small cells as well. Not all, however, of the Fc-rosetting subpopulation was removed this way, suggesting a differential phagocytic susceptibility, particularly amongst the smaller "microglial" cells.

*Rosetting reactions of cultured cells*

*Normal brain.*—Cell preparations from normal brain, over a 2–3 week period, gave rise to a monolayer of elongated cells. Following subculture, however, the cells died and the monolayer was not propagated. Only 10% of the harvested cells rosetted with the different indicator cells, compared with $\sim 50\%$ of the original cell suspension.

*Brain tumours.*—The various glioma-cell preparations (3 specimens) in short-term culture progressively lost their ability to rosette with the various indicator cells; by the third subculture no rosetting cells were detected.

### DISCUSSION

The present study reveals the presence of lymphocytes in some human primary and secondary malignant growths of the brain. The lymphocytes were detected both by histological examination of the tumour specimens (in 6/14 gliomas and in 2 secondary tumours) and by surface-marker rosetting reactions in 5/14 gliomas (36%). This data confirms the previously reported histological findings of perivascular lymphocyte cuffing in human gliomas (Ridley & Cavanagh, 1971; Takeuchi & Barnard, 1976; Stavrou *et al.*, 1977; Palma *et al.*, 1978).

The lymphocytes, when present in the tumour-cell preparations, constituted $< 15\%$ of the total cells isolated; a similar percentage to that found by Wood & Morantz (1979) with enzymatically treated malignant growths of the human brain. No correlation was found, in the samples available, between the pathological type of tumour and the different subsets. The various lymphocyte subpopulations (T lymphocytes predominating) were detected only in those tumour-cell preparations with histologically confirmed prominent lymphocytic infiltrates.

There is a paucity of data in the literature about the various lymphocyte subsets found in tumours of the human central nervous system. Stavrou *et al.* (1977) using tissue sections and a fluorescein isothiocyanate-labelled antiserum raised against human T lymphocytes, found thymus-derived lymphocytes in the perivascular lymphocytic cuffs present in some of the gliomas examined.

Macrophages were also detected and characterized in the human malignant growths of the brain. In contrast to the findings with lymphocytes, histological assessment of tissue sections was found to be an unreliable method for detecting macrophages. Tumour-cell suspensions prepared by mechanical disaggregation, on the other hand, did contain in most preparations a significant number of macrophages (20–60% of the medium–large cells). The majority ($\leqslant 75\%$) of the morphologically obvious macrophages expressed receptors for the Fc portion of IgG. A variable but usually lower percentage expressed receptors for the third component of complement. The percentage of macrophages with surface immunoglobulin was comparable to the Fc-receptor-bearing subpopulation and was probably cytophilic. It was not possible to determine the origin(s) of this cytophilic immunoglobulin. Evidence has accumulated in the literature, however, suggesting that many of the macrophages infiltrating non-malignant lesions in the brain (induced by trauma or viral agents) are of haematogenous origin. Autoradiographic techniques reveal significant numbers of labelled marrow-derived cells or blood monocytes in the damaged brain (Konigsmark & Sidman, 1963; Roessmann & Friede, 1968; Oehmichen & Gencic, 1975; Fujita & Katamura, 1976). It therefore seems probable that many of the tumour-infiltrating macrophages found in the present study were also derived from blood monocytes. Such an origin could explain the presence of cytophilic immunoglobulin on the macrophage surface.

Macrophages have been recently described in both human brain tumours (Wood & Morantz, 1979) and in chemically induced brain tumours in rats (Lantos, 1975; Morantz *et al.*, 1979). Wood & Morantz (1979) using enzyme-dispersed glioma-cell preparations, found a range of values comparable to those in our series, suggesting that the macrophage numbers in our study were not due to selective release of the macrophages by the dispersal technique. The macrophages were assessed morphologically and by the presence of the Fc(IgG) receptor, but as in our rosetting assays, the authors were not able to establish unequivocally the exact nature of some of the Fc-receptor-bearing cells. In contrast to our findings, Wood & Morantz (1979) were not able to detect C3-receptor-bearing macrophages. These authors, on the other hand, used trypsin to prepare their tumour cell suspensions—a procedure known to readily remove C3 receptor from the cell surface (Eremin *et al.*, 1977).

Macroglial cells (Glees *et al.*, 1978) and tumour cells (Kerr & Searle, 1972) have been documented expressing phagocytic activity, and therefore could possibly possess surface receptors for Fc and C3. Our studies, using electron microscopy and astrocyte-specific anti-GFAP, as well as the rosetting reactions, found no evidence to substantiate the presence of malignant cells (astrocytes, oligodendrocytes) rosetting with the different indicator cells in our tumour preparations.

The data obtained in this investigation also shows that cell suspensions from the normal cerebellum and cortex (grey and white matter) of man, obtained either by mechanical disaggregation or by collagenase digestion, are devoid of lymphocytes, but possess cells morphologically like macrophages. These macrophages, like their counterparts in tumour-cell preparations, express receptors (albeit less avidly) for Fc(IgG) and C3, and have surface immunoglobulin (probably cytophilic). We made a similar limited study of 2 normal rat brains and found a population of receptor positive cells.

Autoradiographic studies have provided some evidence that the macrophage-like cells in normal brain probably arise in the marrow (Roessmann & Friede, 1968) and subsequently become circulating blood monocytes (Konigsmark & Sidman, 1963)—not unlike the migratory route of the macrophage into the damaged brain.

Cell suspensions prepared from gliomas and cancer-free brain also contained a third population of cells. This population consisted of small, non-lymphocytic, phagocytic cells expressing receptors for Fc(IgG) and C3. This cell subset may be the resident "microglial" population. Oemichen & Huber (1976) have presented convincing evidence that following trauma to the rabbit brain, glass-adherent, phagocytic cells with Fc(IgG) and C3 receptors accumulate in the wound; some they describe as emigrant blood monocytes and others as proliferative resident microglial cells—differentiated by silver impregnation). Kitamura *et al.* (1977) and Blakemore (1975) also claim to be able to differentiate resident microglial cells from macrophages, by silver staining and the presence of microtubules respectively. There is, on the other hand, equally convincing data from other workers that microglial cells and brain macrophages are either one and the same cell or are derived from the same cell precursor in the brain; the stem cell being initially derived from, and possibly subsequently replenished by, cells of the haematogenous compartment (Lantos, 1975; Das, 1976; Fujita & Kitamura, 1976).

The presence of macrophage-like cells (Fc-receptor-bearing, phagocytic) in gliomas is not entirely unexpected, but the demonstration of such cells as a resident population in human normal cerebral cortex and cerebellum, albeit in a less activated state, is of considerable importance, and points to the existence of a possible immunological defence mechanism, despite the absence of a conventional lymphatic system.

We wish to express our thanks to Mr J. Gleave and Mr A. Holmes of the Department of Neurosurgery, New Addenbrooke's Hospital, for their cooperation. The rabbit anti-human GFAP serum was a gift, and was prepared by Dr M. Raff of the M.R.C. Unit, Department of Zoology, University College, London. This work was supported by the Cancer Research Campaign and a Bebee Foundation grant to Dr Phillips.

### REFERENCES

BLAKEMORE, W. F. (1975) The ultrastructure of normal and reactive microglia. *Acta Neuropathol.*, **6**, 273.

COOMBS, R. R. A., WILSON, A. B., EREMIN, O. & 5 others. (1977) Comparison of the direct antiglobulin rosetting reaction with the mixed antiglobulin rosetting reaction for the detection of immunoglobulin on lymphocytes. *J. Immunol. Methods*, **18**, 45.

DAS, G. D. (1976) Resting and reactive macrophages in the developing cerebellum. An experimental ultrastructural study. *Virchows Arch.* (*B*) *Cell. Pathol.*, **20**, 287.

EREMIN, O., PLUMB, D. & COOMBS, R. R. A. (1976) T and B lymphocyte populations in human normal lymph node, regional tumour lymph node and inflammatory lymph node. *Int. Arch. Allergy Appl. Immunol.*, **53**, 277.

EREMIN, O., KRAFT, D., COOMBS, R. R. A., ASHBY, J. & PLUMB, D. (1977) Surface characteristics of the human K (killer) lymphocyte. *Int. Arch. Allergy Appl. Imminol.*, **55**, 112.

EREMIN, O., COOMBS, R. R. A. & ASHBY, J. (1981*a*) Lymphocytes infiltrating human breast cancers lack K cell activity and show low levels of NK cell activity. *Br. J. Cancer*, **44**, 166.

EREMIN, O., COOMBS, R. R. A., PROSPERO, T. D. & PLUMB, D. (1981*b*) T and B lymphocyte subpopulations infiltrating human mammary carcinomas. *J. Natl Cancer Inst.* (in Press).

FUJITA, S. & KITAMURA, T. (1976) Origin of brain macrophages and the nature of microglia. In *Progress in Neuropathy* (Ed. Zimmerman), Grune & Stratton., **3**, 1.

GLEES, P., SPOERRI, O. & SPOERRI, P. E. (1978) Phagocytic properties of oligodendrocytes. *J. Physiol.*, **278**, 242.

JAMES, K., MCBRIDE, B. & STUART, A. (1977) The macrophage and cancer. *Proc. Eures Symp.* Edinburgh.

KANSKI, A., SPENCER, J. & EREMIN, O. (1981) Sephadex G-10 columns do not retain selectively T or B lymphocyte subpopulations. *J. Immunol. Methods* (in Press).

KERNOHAN, J. W. & SAYRE, G. P. (1952) Tumours of the Central Nervous System. In *Atlas of Tumour Pathology*, Washington: A.F.P.

KERR, J. F. R. & SEARLE, J. (1972) The digestion of cellular fragments within phagolysosomes in carcinoma cells. *J. Pathol.*, **108**, 55.

KITAMURA, T., TSUCHIHASHI, Y., TATEBE, A. & FUJITA, S. (1977) Electron microscopic features of the resting microglia in the rabbit hippocampus, identified by silver carbonate staining. *Acta Neuropathol.*, **38**, 195.

KONIGSMARK, B. W. & SIDMAN, R. L. (1963) Origin of brain macrophages in the mouse. *J. Neuropathol. Exp. Neurol.*, **22**, 643.

LANTOS, P. L. (1975) Macrophages in brain tumours induced transplacentally by N-ethyl-N-nitrosourea in rats: An electron-microscopy study. *J. Pathol.*, **116**, 107.

MEDAWAR, P. B. (1948) Immunity to homologous grafted skin. III. The fate of skin homografts transplanted to the brain, to subcutaneous tissue, and to the anterior chamber of the eye. *Br. J. exp. Pathol.*, **29**, 58.

MORANTZ, R. A., SHAIN, W. & GRAVIOTO, H. (1978) Immune surveillance and tumours of the nervous system. *J. Neurosurg.*, **49**, 84.

MORANTZ, R. A., WOOD, G. W., FOSTER, M., CLARK, M. & GOLLAHON, K. (1979) Macrophages in experimental and human brain tumours. *J. Neurosurg.*, **50**, 298.

OEHMICHEN, M. & GENCIC, M. (1975) Experimental studies on the kinetics and functions of the mononuclear phagocytes of the central nervous system. *Acta Neuropathol.*, **6**, 285.

OEHMICHEN, M. & HUBER, H. (1976) Reactive microglia with membrane features of mononuclear phagocytes. *J. Neuropathol. Exp. Neurol.*, **35**, 30.

PALMA, L., DILORENZO, N. & GUIDETTI, B. (1978) Lymphocytic infiltrates in primary glioblastomas and recidiuous gliomas. *J. Neurosurg.*, **49**, 854.

PHILLIPS, J. P., EREMIN, O. & GLEAVE, J. (1979) Proceedings of the Society of British Neurological Surgeons (Abstract), *J. Neurol. Neurosurg. Psychiat.*, **42**, 967.

RAFF, M. C., FIELDS, K. L., HAKOMORI, S-I., MIRSKY, R., PRUSS, R. M. & WINTER, J. (1979) Cell-type specific markers for distinguishing and studying neurons and the major classes of glial cells in culture. *Brain Res.*, **174**, 283.

RHODES, J. (1980) Resistance of tumour cells to macrophages: A short review. *Cancer Immunol. Immunother.*, **7**, 211.

RIDLEY, A. & CAVANAGH, J. B. (1971) Lymphatic infiltration in gliomas: evidence of possible host resistance. *Brain*, **94**, 117.

ROESSMANN, V. & FRIEDE, R. L. (1968) Entry of labelled monocytic cells into the central venous system. *Acta Neuropathol.*, **10**, 354.

SCHEINBERG, L. C., EDELMAN, F. L., LEVY, W. A. (1964) Is the brain an "immunologically privileged site?" I. Studies based on intracerebral tumour sensitized hosts. *Arch. Neurol.*, **11**, 248.

SCHEINBERY, L. C., LEVY, A., EDELMAN, F. Is the brain "an immunologically privileged site"? II. Studies on induced host resistance to transplantable mouse glioma following irradiation or prior implants. *Arch. Neurol.*, **13**, 283.

STAVROU, D., ANZIL, A. P., WERDENBACH, W. & RODT, H. (1977). Immunofluorescence study of lymphocytic infiltration in gliomas. *J. Neurol. Sci.*, **33**, 275.

TAKEUCHI, J. & BARNARD, R. O. (1976) Perivascular lymphoctyic cuffing in astrocytoma. *Acta Neuropathol.*, **35**, 265.

UNDERWOOD, J. C. E. (1974) Lymphoreticular infiltration in human tumours. Prognostic and biological implants. A review. *Br. J. Cancer*, **30**, 538.

VOSE, B. M. & MOORE, M. (1979) Supressor cell activity of lymphocytes infiltrating human lung and breast tumours. *Int. J. Cancer*, **24**, 574.

WOOD, G. & MORANTZ, R. A. (1979) Immunohistologic evaluation of the lymphoreticular infiltrate of human central nervous system tumours. *J. Natl Cancer Inst.*, **62**, 485.